# Digital Lifestyle Interventions for Young People With Mental Illness: A Qualitative Study Among Mental Health Care Professionals

**DOI:** 10.2196/53406

**Published:** 2024-06-05

**Authors:** Chelsea Sawyer, Rebekah Carney, Lamiece Hassan, Sandra Bucci, John Sainsbury, Karina Lovell, John Torous, Joseph Firth

**Affiliations:** 1 Division of Psychology and Mental Health University of Manchester Manchester United Kingdom; 2 Greater Manchester Mental Health NHS Foundation Trust Manchester United Kingdom; 3 Division of Nursing, Midwifery & Social Work University of Manchester Manchester United Kingdom; 4 Beth Israel Deaconness Medical Centre Harvard Medical School Boston, MA United States

**Keywords:** digital health, behavior change, mental health care professionals, physical health, lifestyle intervention, qualitative, thematic analysis, service optimization, mobile phone

## Abstract

**Background:**

Given the physical health disparities associated with mental illness, targeted lifestyle interventions are required to reduce the risk of cardiometabolic disease. Integrating physical health early in mental health treatment among young people is essential for preventing physical comorbidities, reducing health disparities, managing medication side effects, and improving overall health outcomes. Digital technology is increasingly used to promote fitness, lifestyle, and physical health among the general population. However, using these interventions to promote physical health within mental health care requires a nuanced understanding of the factors that affect their adoption and implementation.

**Objective:**

Using a qualitative design, we explored the attitudes of mental health care professionals (MHCPs) toward digital technologies for physical health with the goal of illuminating the opportunities, development, and implementation of the effective use of digital tools for promoting healthier lifestyles in mental health care.

**Methods:**

Semistructured interviews were conducted with MHCPs (N=13) using reflexive thematic analysis to explore their experiences and perspectives on using digital health to promote physical health in youth mental health care settings.

**Results:**

Three overarching themes from the qualitative analysis are reported: (1) motivation will affect implementation, (2) patients’ readiness and capability, and (3) reallocation of staff roles and responsibilities. The subthemes within, and supporting quotes, are described.

**Conclusions:**

The use of digital means presents many opportunities for improving the provision of physical health interventions in mental health care settings. However, given the limited experience of many MHCPs with these technologies, formal training and additional support may improve the likelihood of implementation. Factors such as patient symptomatology, safety, and access to technology, as well as the readiness, acceptability, and capability of both MHCPs and patients to engage with digital tools, must also be considered. In addition, the potential benefits of data integration must be carefully weighed against the associated risks.

## Introduction

### Background

People with “severe mental illness” (SMI), such as schizophrenia, bipolar disorder, and associated psychotic or mood disorders, experience poorer physical health outcomes, which negatively affects their well-being across the life course and reduces life expectancy by up to approximately 15 years [1-3]. To reduce health disparities, it is crucial to adopt a preventative approach and intervene early. Adolescence or young adulthood presents a key opportunity as this is when most enduring mental health conditions are first diagnosed [4]. Young people with SMI and those at risk of SMI exhibit signs of poor cardiometabolic health and are more likely to engage in behaviors that are detrimental to their physical health; yet, much of this risk is modifiable [4,5].

Mental health care professionals (MHCPs) play a crucial role in supporting the mental and physical health needs of people with psychosis. However, MHCPs face significant barriers to delivering physical health interventions in practice [6]. This includes inadequate time and training in delivering evidence-based physical health interventions, difficulty reaching people in rural or remote areas, financial implications of delivering face-to-face interventions (particularly one-to-one), and limited National Health Service (NHS) resources for implementation [6].

Given these barriers and the increasing demand on the NHS, there is a growing focus on digital lifestyle interventions (DLIs), for example, using smartphones and websites to provide low-cost, scalable, and flexible interventions to promote healthier lifestyles [6,7]. The delivery of DLIs will require behavior changes among MHCPs. One model that can explain behavior change is the Capability, Opportunity, and Motivation–Behavior (COM-B) model [8]. According to this model, MHCP capability and opportunity to perform the behavior will influence their motivation to use DLIs and impact their delivery of DLIs in mental health care (MHC) settings. Capability refers to whether a person has the psychological (knowledge) or physical (skills) capability to perform the behavior. Alongside capability, an individual must have the opportunity to perform the behavior, and this refers to both physical (this includes the environment where the behavior will be performed and resources such as money and time) and social (the behavior of others) opportunity. Both reflective (reflective processes such as beliefs, goals, and values) and automatic (habitual and emotional responses) motivation also influence our behavior. The COM-B model can be used to inform future interventions [8].

Previous research suggests that MHCPs see the benefits of physical activity interventions [9]. However, MHCPs report barriers to implementation such as concerns about patient motivation and safety and logistical concerns on behalf of the patient, such as having equipment, clothes, and space. MHCPs have also reported personal barriers such as low confidence and capability to deliver interventions, lack of time and resources, and the belief that MHC should be a priority [10].

It is likely that MHCP attitudes toward and perceived barriers to using DLIs in MHC settings will vary from those for in-person interventions. According to actor-network theory, technology is not simply a tool or passive instrument that humans use to accomplish their goals [11]. Instead, technology can shape human behavior by creating new opportunities, alleviating constraints, and providing affordances that shape the way in which people think, communicate, and interact. Previous research has found that MHCPs believe that digital tools that support patient self-management would change their own roles and responsibilities [12]. Numerous studies have shown that, while MHCPs see the potential benefits, they are concerned about issues of liability, harm to patients, and lack of training regarding using DLIs [12-14]. As the MHCP role primarily focuses on treating mental health difficulties [15], it is important to explore and compare MHCP beliefs about using digital health for managing symptoms versus delivering lifestyle interventions.

### Objectives

Therefore, this study aimed to explore MHCP perspectives, including barriers to and facilitators of using DLIs in MHC settings, with a particular focus on young people. These insights will provide key considerations for the implementation and use of DLIs in MHC settings.

## Methods

### Study Design

A mixed methods design was used, including a web-based survey and qualitative interviews, to examine the attitudes of MHCPs toward digital health in young people’s MHC. An overview of the aligned findings of the combined survey and interview components of the project has been presented elsewhere [15]. While the previous mixed methods analysis provides a foundation for the research presented in this paper, this paper focuses on presenting the results of an in-depth qualitative examination of all the interview data, presenting the subjective experiences and perspectives of MHCPs regarding digital technology. Using a reflexive thematic analysis, we offered a more comprehensive understanding of subjective factors affecting the barriers and implementation of DLIs in clinical practice beyond the scope of the previous descriptive results to present useful recommendations for facilitating uptake of novel technology in health care settings.

The inclusion criteria were NHS MHCPs (1) working with young adults aged 16 to 35 years with mental illness, including specialist mental health services or in the context of broader primary care, and (2) working with young adults with mental illness for at least 6 months. The exclusion criteria were MHCPs working primarily with eating disorders due to differing treatment needs on nutrition and exercise [16-18]. The COREQ (Consolidated Criteria for Reporting Qualitative Research) checklist was used to ensure a comprehensive and explicit report of the interview process (Multimedia Appendix 1).

### Participants

All 13 participants were MHCPs working within NHS services with young adults aged 16 to 35 years, including specialist mental health services. Purposive sampling was used to recruit potential participants of a variety of occupational backgrounds and years of experience. Participants were recruited through emailed flyers. While interview participants were not reimbursed or rewarded for taking part in the interview, survey respondents were offered the choice to enter a prize draw to win a £50 (US $62.62) voucher. It was made clear to participants that completing the interview did not increase their chances of winning.

### Data Collection

#### Overview

Semistructured interviews were conducted remotely using Microsoft Teams (Microsoft Corp) and audio recorded with participant consent. The interviews lasted 26 to 79 minutes and followed a topic guide (Multimedia Appendix 2) developed with input from our Patient and Public Involvement group and research team. In total, 2 researchers (CS and JF) conducted the interviews, which consisted of questions about participants’ experience using digital health, the potential use of mobile health and DLIs in MHC, barriers to integration and use, and ways to boost engagement. Interview guides were flexible, using prompts and open questions to encourage participants to talk in depth about their experiences. All interviews were recorded and transcribed verbatim. Participants were assigned pseudonyms to maintain anonymity.

#### Data Analysis

Interviews were analyzed using a reflexive thematic approach [19-21]. Reflexive thematic analysis involves the researcher reflecting on how their experiences, personal assumptions, and background shape their analysis and interpretation of the data [20]. An inductively orientated experiential approach underpinned by critical realism was used [20]. This means that our themes were generated from the interviewees’ direct experiences and observations while also recognizing that their understanding of reality is shaped by social and cultural factors. Critical realism was used as it allows the researcher to analyze participants’ experiences while allowing the analysis to be informed by theory. The COM-B model of behavior change [21], which proposes that behavior is defined by our capability, opportunity, and motivation, was used as a theoretical underpinning and a prespecified area of interest. The model was used to identify potential barriers to the implementation of DLIs for young people with mental health conditions and any potential solutions to overcome these barriers.

Thematic analysis is a systematic approach whereby patterns and common themes are identified to describe a data set and understand a phenomenon [19,20]. The 6-phase guidelines by Clarke and Braun [20] were used to guide the analysis. These phases are recursive: (1) transcripts were read and reread so that the researcher (CS) could become familiar with the data, (2) systemic line-by-line coding was conducted to identify common features in the data, (3) codes were reviewed to determine themes, (4) themes were reviewed by 3 researchers (JF, CS, and LH) for homogeneity and heterogeneity to ensure that they were distinctive and coherent, (5) themes were defined and names were generated, and (6) findings were reported.

A primarily inductive approach was adopted with the interviews, but a deductive approach was taken when examining the barriers and facilitators informed by the COM-B model (based on previous research). Interview extracts related to barriers to and facilitators of implementing DLIs were mapped to the components of the COM-B model, whereas an inductive approach was taken for the remaining data. At the time of analysis, the researcher was not familiar with the current literature on digital health and in particular in the context of mental health, allowing them to analyze the data without preconceived themes or experiences.

To reduce the risk of bias, all researchers were involved in the analysis through regular meetings to discuss codes and themes. Themes and subthemes were generated and finalized using the NVivo software (version 12; QSR International) and MindView (version 7.0; Matchware). The team discussed the themes until consensus was reached within the team. During manuscript writing, subthemes with overlap were combined to avoid repetition. The final theme structure presented in the manuscript was reviewed and agreed upon by all coauthors.

#### Reflexivity

The two researchers who conducted the interviews (JF and CS) do not work in the NHS and made this clear to interviewees who were NHS employees. Both researchers have experience interviewing people on a variety of sensitive topics (self-harm, cancer [CS], and mental health [JF and CS]). All authors have an interest in digital health and promoting physical health in MHC settings. First author CS personally uses digital health apps. These views and experiences may have influenced our analysis; therefore, author CS kept a reflexive journal throughout the study. The author routinely reflected in the journal during data collection and analysis to reduce the possibility of their personal experiences and beliefs biasing their interpretations. A potential influencing factor in the interviews could be attributed to age. Some of the interviewees remarked that they did not have the same familiarity with apps as the interviewer (CS); this assumption could have influenced the views and opinions that participants expressed to this author.

Due to the COVID-19 pandemic, the interviews were conducted remotely using Microsoft Teams. Most participants joined the interviews from their own homes, providing a private setting that potentially fostered comfort and openness. However, working from home could have affected their work mindset and introduced distractions, such as pets, deliveries, or background noises. During an interview held in a private room at an interviewee’s workplace, a team member interrupted the participant. While this interruption may have had an impact, it did not seem to alter their perceived barriers, and they continued discussing barriers, including those related to NHS staff, possibly influenced by their senior role. The remote format of Microsoft Teams interviews might have resulted in the interviewer missing out on subtle body language and facial responses, especially in the case of one participant who opted to keep their camera off. Nonetheless, Microsoft Teams provided flexibility, enabling participants to join at their preferred times.

### Patient and Public Involvement

The overall study protocol had patient and carer involvement to ensure that all materials were appropriate and the content discussed about patients was appropriate and meaningful. The topic guide was developed using lived experience input. RC is a research fellow at a research unit embedded within clinical services at the Greater Manchester Mental Health NHS Foundation Trust (JUICE Youth Mental Health Research Unit). JUICE consists of academics as well as current practicing clinicians such as ward managers, lead psychiatrists, therapists, physiotherapists, dietitians, occupational therapists, experts by experience, and carers. A weekly consultation is held on the Child and Adolescent Mental Health Services inpatient units, where the topic guide was discussed.

### Ethical Considerations

Ethics approval was granted by the University of Manchester Research Ethics Committee (2020-10603-17104) and the Health Research Authority (288734). Participants were briefed on the purpose of the study, and written informed consent was obtained.

## Results

### Participant Characteristics

A total of 13 MHCPs were recruited from various MHC settings and roles, including MHC and research nurses (n=4, 31%), trainee psychiatrists (n=2, 15%), support workers (n=2, 15%), occupational therapists (n=1, 8%), physical health support workers (n=1, 8%), service managers (n=1, 8%), operational leads (n=1, 8%), and trainee advanced practitioners (n=1, 8%). Of the participants, 85% (11/13) were female, and 100% (13/13) were White British, with experience varying from at least 6 months to 20 years working in MHC service.

### Analysis

#### Overview

**Figure 1:**
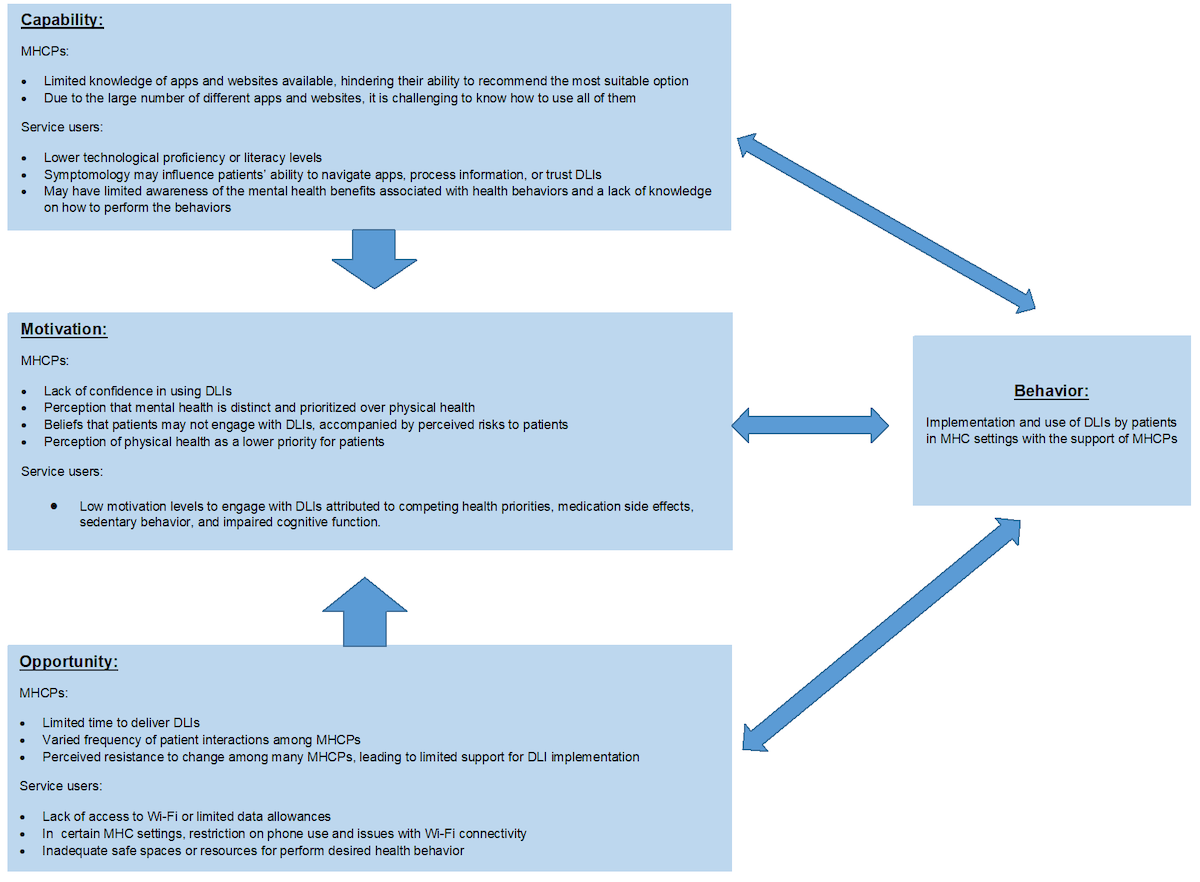
Barriers to implementation of DLIs in MHC using the COM-B model_v2. COM-B: Capability, Opportunity, and Motivation–Behavior; DLI: digital lifestyle intervention; MHC: mental health care; MHCP: mental health care professional.

Three main themes were identified: (1) motivation will affect implementation, (2) patients’ readiness and capability, and (3) reallocation of staff roles and responsibilities. The themes and subthemes are described in Multimedia Appendix 3. Figure 1 presents how our findings also fit with the domains of the COM-B model [8].

#### Theme 1: Motivation Will Affect Implementation

##### Overview

MHCP and patient motivation was perceived as the largest potential barrier to implementing DLIs in MHC settings. All interviewees felt that it would be important to emphasize the value and benefit of DLIs to patients, whereas acknowledging their potential risk is crucial for implementing DLIs in MHC settings:

I think some of them [MHCP], don’t necessarily promote the apps, erm, because of the motivation...it can be quite problematic getting them [patients] to engage.

INT5

##### Subtheme 1: Individuals Are Motivated but Others Are Resistant

Most participants had a positive personal view of using digital health in MHC, in particular for young adults. However, some interviewees said that, although they were personally motivated to introduce and implement DLIs in the context of MHC delivery, their colleagues were reluctant to change, which posed a barrier to rolling out DLIs. MHCPs who are unaware of the benefits of DLIs or, for example, who are accustomed to established ways of working, were perceived by interviewees to be more resistant to adopting new practices:

...lots of people don’t like change do they, and I think whenever you try and do anything new they’ll be somebody that, will have something to say about it. I can’t think of any specific kind of negative view of it, other than that, that just, traditional view that, oh it won’t work, they won’t use it.

INT10

Furthermore, several participants expressed concerns about time constraints and competing priorities faced by MHCPs. With an already demanding workload, interviewees said that MHCPs tend to prioritize mental health–focused care over physical health approaches or interventions. Interestingly, most people felt that their role was not best suited to the introduction of DLIs due to limited interactions with patients compared to their care coordinators despite being supportive of DLIs and their benefit more generally:

...it’s [digital interventions] probably one of those things it’s quite a good idea for the whole team to have an awareness of, but maybe, erm, you know, particularly care coordinators who are having the most contact with service users.

INT9

##### Subtheme 2: Patients Have Other Priorities

Interviewees perceived low motivation among young adults as a common barrier to engaging with DLIs. Low motivation was often attributed to medication side effects, sedentary behavior, and impaired cognitive function. Many interviewees perceived physical health to be a low priority for patients and that patients’ main priority during their involvement with an MHC team was their mental health:

I think it depends on where they’re at within their illness and how engaged they are with treatment and especially during those initial phases, it can be quite problematic, erm, and getting them to...engage.

INT5

Some interviewees felt that it was important to highlight the link between patients’ physical and mental health and how changes to behavior could lead to changes in medication or treatment as well as improving physical and mental health:

Well talk about how, erm, maybe weight changes affect their mental health, how their medication has, the amount of medication that they’ve had has changed, how their, erm, you know, their diabetes diagnosis was reversed because they engaged in exercise and speak to them about what significance it would be to them, it’s not about a size 8 jeans, it’s about, I don’t need to take as much Clozapine, and plus if I don’t take as much Clozapine at night, then in a morning I’m not as knackered.

INT5

Interviewees also felt that the service setting might influence the uptake of DLIs. For example, young adults in inpatient settings may be hesitant to change due to the various restrictions in place, such as limiting takeaways or access to unhealthier food. DLIs that provided young adults with something such as promoting physical activity in inpatient settings were viewed as acceptable by interviewees. However, DLIs viewed as restrictive, such as those concerning diet or smoking, were assumed as not being well received by patients:

...inpatient mental health unit, erm, you’ve had so many bits of your identity taken away from you, in terms of like being able to access outside, and, I think, when you do try and have those conversations about, you know, well try and eat a bit healthier, it’s gotten very angry really quickly because there’s been so much of their liberties taken away, the fact that we try and take away the little things that they do enjoy like, staying in bed, erm, eating junk food, smoking, it, it, yeah, it can be quite a difficult subject.

INT11

Therefore, these preassumptions regarding what types of DLIs young adults are resistant to may reduce MHCP enthusiasm for undertaking the actions required to implement specific DLIs in inpatient settings. One MHC service that several MHCPs felt could work well to integrate DLIs in was early intervention services:

I think the young, younger people are more likely to use apps and You know when people first present to services, such as early intervention team. Uh, I think that would would work very, very well.

INT1

##### Subtheme 3: DLIs Need to Be Intuitive and Engaging

To overcome the perceived low motivation of patients to engage with DLIs, MHCPs felt that DLIs need to capture patients’ attention and be engaging, which involved being visually appealing, interactive, and user-friendly, particularity due to the patients’ age. Gamification, linking changes in behavior to patient-valued outcomes, and intuitive interfaces were perceived as being important in promoting engagement. Simple designs and usability were considered key, ensuring that both MHCPs and patients can navigate through the intervention:

...if you’re focussing on young people, I think, I don’t really see many barriers, if it’s free, and, you know it’s easy to use, I think it’s just, it’d just be about that initial engagement, that initial kind of, them trying and it being good enough to keep them, er, interested.

INT10

...anything that’s simple, straightforward and just easy to use would be probably the best starting point for now, for us [Mental Health are professionals].

INT12

I've never used gamified apps to be honest up, but yeah, it sounds great, It will make the younger people engage.

INT2

Several MHCPs shared the perspective that involving young adults with a wide spectrum of mental health conditions and literacy skills in the app design process is crucial to ensure both intuitiveness and engagement for patients:

...the important thing would be that if you were gonna design an app to, to genuinely have young people with a variety of mental health conditions, neurodevelopmental disorders, etc, all having input on what it looks like, how it works and how you engage with it.

INT13

#### Theme 2: Need to Consider Patients’ Readiness and Capability to Use Digital Health

##### Subtheme 1: Patient Safety

A prominent theme was the need for patient benefit from digital health to outweigh potential risks or harm. Interviewees expressed the need to consider each patient and when was best to introduce DLIs. Initially, interviewees focused on what MHC settings would be the most suitable to introduce DLIs, with mixed views on the appropriateness of implementing DLIs in inpatient settings. On further reflection, the severity of the patients’ symptoms rather than the MHC setting was considered most important when deciding on an appropriate time to introduce patients to DLIs:

...people with schizophrenia aren’t unwell constantly, so you would use it and if they started to become paranoid or unwell, that’s probably when you’d be able to just say, let’s just remove it.

INT12

Concerns for patient safety were raised if DLIs required the young person to self-monitor physical activity or health data. For example, there were concerns that patients might misunderstand and assume that their MHC team was also monitoring their data, potentially leading them to not inform their MHC team of important changes. Alternatively, if health data were integrated and monitored by their MHC team, there were concerns that important data such as irregular heartbeat or reduced smoking while on clozapine could be missed, which could lead to harm:

I’m just thinking about difficulty I’d, I’d, feel terrible. If I had somebody on my caseload as a care coordinator and this information was there and I didn’t pick up on it and I didn’t notice that and then something happened, it’s. Then there’s a kind of risk factor there of Case negligence, maybe potentially, and who’s going to be overseeing that.

INT1

Interviewees were also concerned that DLIs could worsen young adults’ mental health symptoms, including paranoia, particularly for those who used phones or wearable devices. MHCPs articulated concerns that the tracking of patients’ location and behavior via these wearable devices and phones could intensify patients’ anxieties regarding perceived surveillance:

Consideration for people who could be psychotic, paranoid, suspicious, you know, and whether that might increase some of their Symptomatology, illusions or paranoia. You know if they were wearing a watch. For instance, knowing that I had access to that information.

INT1

MHCPs expressed concerns about potential risks that DLIs could pose regarding social interactions for young adults with psychosis. They were worried about inaccurate or harmful advice, exploitation, or negative interactions. Therefore, ensuring monitoring and moderation of social interactions within DLIs was necessary. However, interviewees also recognized the benefits of social support in boosting engagement:

...you would need some level of moderation in that community, ’cos you don’t want unhelpful comments and views and, and unfortunately with online systems you get a lot of that. erm, because people are anonymous...there could be like a positive element of that and it could increase engagement.

INT13

##### Subtheme 2: Patients’ Capability and Opportunity to Use Digital Technology

Digital health was perceived to be acceptable in MHC, especially among younger patients who were likely to have higher smartphone use. However, symptoms (eg, paranoia), reduced cognitive abilities (partly due to medication), and limited technological skills were seen as barriers to patients using digital technology:

A lot of the younger people are, erm [have technical skills], but, but also even, even the younger people when they’re unwell, they have information processing problems.

INT6

Some MHCPs commented that paranoia may also hinder engagement. To address this, collecting minimal personal data through apps or websites was recommended:

I think just, the simpler the better maybe, you know, not having to gather as, as much information, just going off, you know, erm, individuals who, who can paranoid or, or the barriers, I think it, just something that’s simple, that’s easy, you know, you’re not having to put a lot of data in.

INT7

Access was a potential barrier as some patients may lack access to phones, data, or apps, especially during periods of psychosis or as inpatients due to restrictions or poor Wi-Fi connections. Interviewees suggested that data-free apps that synchronize to Wi-Fi or apps with lower data requirements might overcome this barrier. To reduce the digital divide, interviewees said that services (ie, the NHS) should provide smartphones, pay for subscription costs or data network charges, or provide wearables to ensure that recommended DLIs can be accessed fairly:

If something’s gonna be effective and there’s evidence base into it, I don’t think that people should [pay], if it’s about health and it’s actually gonna reduce our costs in the long term, then it should be free.

INT13

#### Theme 3: Integrating Digital Health Will Require the Reallocation of Staff Roles and Responsibilities

##### Subtheme 1: Technology Changes Our Roles and Responsibilities

Despite attitudes being largely positive, there were differences in interviewees’ perspectives on the impact that implementing DLIs into routine care would have on MHCP workload. Some interviewees who felt that their role should mainly focus on the mental health needs of a patient believed that DLIs would increase their workload. They suggested that the care coordinator (case manager) role would be best placed to implement DLIs as they have more patient contact and involvement and, therefore, have the time and a preexisting relationship:

...particularly care coordinators who are having the most contact with service users.

INT9

In contrast, interviewees in senior or physical health–focused professions viewed digital health as a way to reduce staff workload and enable frequent physical health monitoring (eg, remote blood pressure monitoring), resulting in better patient care; these interviewees believed that it was everyone’s role and responsibility to implement DLIs in MHC:

...anything that’s from that, the core components of the physical health check that they can input, would save everybody a lot of time and, erm, effort and money and it would also make it much more up to date.

INT10

Interviewees expressed concerns that the use of DLIs in MHC could affect their interactions with patients. Some interviewees believed that building a strong relationship with patients was crucial and DLIs entail a loss of face-to-face nuances, resulting in difficulties detecting physical symptoms, which in turn would be detrimental to patients’ mental health. While some interviewees felt that DLIs may lead to people “becoming more isolated” (INT3), others felt that they would improve access to services for patients, particularly those with social anxieties and those who, due to their younger age, are more comfortable interacting digitally:

...with our cohort who might socially find things challenging and difficult actually a screen is quite familiar to them, so they will often prefer that.

INT8

##### Subtheme 2: MHCPs Will Need to Acquire Additional Skills

Interviewees said that staff involvement was crucial for successful implementation of DLIs in MHC settings. However, interviewees recognized that not all staff members have the necessary skills or knowledge to do this. Tailored training, including interactive sessions and MHCPs using the apps themselves, was recommended to enhance confidence, motivation, and psychological capability to use and recommend digital technology in clinical settings:

There's always room for training I find that interesting to go through it and if I was clinician, finding out what apps there are out there, how we can use them, how we can recommend.

INT1

One interviewee also felt that digital health education could be provided in health care degrees such as nursing and occupational therapy. Several MHCPs lacked awareness of available apps or websites. To address this, interviewees suggested that the NHS could provide a list of approved apps, improving trust and credibility while improving their knowledge and reducing guilt or personal blame in case of adverse events. Opinions differed on recommending apps without previous experience. Some expressed concerns about patient safety when recommending potentially ineffective or harmful apps, such as exercise apps that result in injury, whereas others felt comfortable if the apps were available to the general population:

...some people, if it’s not NHS approved, might be a bit more nervous. I think that’s a thing with the YouTube videos as well, like if it’s just a person who’s put together a, an exercise for somebody to follow, and it’s not to do with the NHS, I think it does make people a bit more nervous about engaging people in that, but I personally have done, if, if I feel confident.

INT11

##### Subtheme 3: Who Is Responsible for Managing the Risk?

A concern regarding the implementation of DLIs was the issue of responsibility. All interviewees questioned who would be responsible if patients experienced negative outcomes as a result of using digital technology in the context of their health care. A particular concern was related to data monitoring, such as what data MHCPs should have access to and who should monitor them (patients or MHCPs). Interviewees expressed concerns about who was responsible for the oversight of digital data collection, especially if fluctuations in health or changes in behavior were missed. Interviewees wondered about their potential liability and described the guilt that they would feel if important changes in mental or physical health were missed. Some MHCPs strongly believed that data should never be integrated into NHS systems but patients could share and discuss their health with MHCPs if desired, empowering patients to take responsibility of their health:

I was thinking about the patient doing it for themselves rather than anybody having anybody sitting behind the scenes monitoring it.

INT2

On the other hand, some interviewees who routinely collected physical health data felt that, if DLIs collected data without MHCPs acknowledging or providing feedback in response, this could demotivate younger patients to use apps that track or record behavior:

I think for this kind of age group and even older, you need that, well done, you’re doing well there, and not, relentless, I think having a barrier would be if there wasn’t any kind of short-term goals that you could say, right we’re making progress here, we’re doing well, or this is what you need to work on.

INT8

...like the feedback, you know, ’cos a lot of the young people that, that we work with on the wards, they really, are seeking time with people and if you can provide that time that’s quite focused on something and provide lots of positive reinforcement if something’s gone really well, I think it might motivate people to, to get sustained use from an app like that, you probably need somebody who’s on your side and really supporting you to use it well.

INT11

In addition, interviewees believed that young adults with mental health conditions may need support and advice from MHCPs in cases in which they implemented minor changes but did not observe any discernible outcomes, for example, if they made changes to their diet but did not lose weight. To overcome the burden of data monitoring and potential liability, one interviewee suggested implementing automated systems that notify clinicians of changes in heart rate, smoking behavior, and so on:

I prefer the idea of, I think it’s a fantastic idea for them [young adult service users] to go away and when they come back and you say, your weight, you’ve been putting weight on, let’s have a look on your app what you actually have been eating, so you can have those sort of discussions with them.

INT6

## Discussion

### Principal Findings

The aim of this study was to explore MHCP perspectives, including barriers to and facilitators of using DLIs to support young people with mental illness. To our knowledge, this is the first study to explore MHCP views on using and integrating digital health in MHC for young people. Overall, MHCPs felt that digital health care is acceptable when delivered alongside face-to-face care and has the potential to enhance the current care that patients receive. However, they also identified barriers to implementation, including staff and patient motivation and capability to deliver or use DLIs, concerns regarding patient safety, the digital divide, and the privacy of data.

### Relevance to Previous Research and Existing Theory

#### Overview

Similar to previous research on lifestyle interventions [9,10,22], MHCPs expressed concerns with patient motivation and safety as well as time constraints and staff motivations and capability to deliver interventions. Notably, there were differences in concerns about resource availability as, while there were concerns about insufficient phone data interfering with DLIs, a lack of other resources (such as home exercise equipment, healthy eating ingredients, or staffing and clothing [9,22]) was not commonly raised as an issue in this study. The remote nature of DLIs raised concerns about missing important data, and key considerations for implementation included ensuring the credibility and trustworthiness of the apps or websites used in DLIs, prioritizing patient safety, and effective data monitoring.

In some cases, MHCPs may differentiate between the promotion of physical health and their core responsibilities. A decreased enthusiasm among MHCPs for addressing physical well-being may be tied to factors such as apprehension toward assuming personal responsibility and diminished patient motivation [10].

Our findings were in line with actor-network theory [11]—MHCPs did not perceive the implementation of DLIs as an additional resource to use. Instead, they felt that the implementation of DLIs would change their roles, bring new risks to patients, and affect rapport. However, a recent study exploring the views of patients with SMI on digital health found that they also believed that such technologies could change the relationship with MHCPs but in a positive way by empowering them to manage their health and providing a source of help other than their health care providers [18,23]. They also valued the ability to self-monitor and share their progress or behavior with their MHCPs to obtain additional support or positive feedback [23]. These results are also in line with those of broader digital health implementation studies suggesting a need for support for patients and clinicians as well as systems-related issues such as regulation, workflow, and safety [24]. Our findings also fit with the domains of the COM-B model [8] (Figure 1). Barriers identified from the interviews related to capability, opportunity, and motivation, along with potential solutions to overcome these barriers generated by the researchers, are presented in Table 1. The potential solutions were then mapped to potential intervention functions (broad categories to change behavior), and these potential barriers and solutions are discussed in more detail in the following sections.

**Table 1:** Potential problems and solutions for the implementation of digital lifestyle interventions (DLIs) in mental health care settings.

Domain and problem			Solution		Intervention function
**Capability**					
	Patients may have poorer digital literacy and skills to use technology or phones (perceived as less of an issue for younger populations)	Instructions on how to effectively use phones and apps		Education	
	Symptoms that may limit patients’ cognitive ability to use digital technology (eg, side effects of medication)	Introduce DLIs only when patients have the mental capacity to consent		Restriction	
	MHCPs^a^ do not know which apps are available and effective and how to use them	Training on how to find and use apps and knowledge sharing within clinical teams		Training or education	
**Opportunity**					
	Patients not having access to phones, internet, or data	Provision of phones, payment for data and wearables, and use of apps that are usable offline and resynchronize or update once connected to Wi-Fi or data		Enablement or environmental restructuring	
	Cost of wearables, data, and app subscriptions	Provision of wearables, NHS^b^ covering the cost of app subscriptions, and having an iPad or device that can be used by an entire inpatient ward or service		Enablement or environmental restructuring	
	Restrictions (eg, no phones and no space) and restricted use or functionality when in inpatient units	Having a shared device on the ward or having supervised access to use the app and being shown how to use it before discharge		Service provision	
	MHCPs do not have the time to deliver interventions	Peer coaches or digital navigators to help patients install apps and deal with issues		Environmental restructuring	
	Integrating health data to ensure patient safety	Automated notifications if there are high risks or behavior changes that need to be addressed (ie, changes in smoking on clozapine)		Enablement	
	Secure storage of data	Improvement in the current technology infrastructure of the NHS to allow app data to be securely stored on the system		Enablement	
**Motivation**					
	Patients having low motivation	To boost motivation, use rewards, games, self-monitoring of behaviors or health outcomes, and provision of feedback		Incentives	
	MHCPs lack confidence with digital technology	Training and education and having regular drop-in clinics to problem solve any issues		Training	
	MHCPs’ competing priorities (focus on treating mental health)	When implementing DLIs, services will need to consider which MHCP roles would be best to implement this and could use individual or service-based targets regarding delivering DLIs		Incentives or coercion	
	MHCP beliefs that patients do not want to change behavior	Involve patients in the integration of DLIs; this will demonstrate that patients are willing to use DLIs		Persuasion	
	MHCPs do not see the benefit of digital interventions	Educating MHCPs on the benefit of treating physical and mental health together and how digital health can play a role in this		Education	

^a^MHCP: mental health care professional.

^b^NHS: National Health Service.

#### Capability

MHCPs voiced uncertainties about their own confidence and ability to effectively deliver DLIs. According to our previous work, MHCPs have limited opportunities to use DLIs in their current role [15], and this is consistent with current literature [13,25]. This may have led MHCPs to perceive a lack of psychological capability (knowledge) to recommend and use apps. Therefore, appropriate training is required, such as interactive training sessions that allow MHCPs to trial various apps and websites, thereby boosting their self-efficacy (the belief in their ability to perform the behavior) and motivation [26]. Alternatively, some MHCPs suggested that participating in DLIs themselves could enhance their motivation. This notion is supported by recent research demonstrating that MHCPs who actively participated in exercise were more inclined to encourage its adoption among inpatients [10].

A lack of knowledge of apps available was perceived as a barrier. MHCPs felt that receiving a list of approved apps to recommend would reinforce the credibility and trustworthiness of the apps, remove personal liability, and improve their knowledge about what is available. Interestingly, very few MHCPs were aware of organizations such as the Organisation for the Review of Care and Health Apps or SilverCloud, which provide a list of NHS-approved apps. Awareness of approved and endorsed apps could reduce the burden on MHCPs in selecting appropriate apps for patients. For example, the MINDapps database allows users to search for mental health apps using specific criteria and provides a description, a rating, and reviews of each app.

MHCPs emphasized the need for considering the clinical presentation of young adults with a mental health condition before the initiation of DLIs and continuously monitoring it should any clinical deterioration occur. They stressed the importance of evaluating symptom severity and diagnosis, with particular care taken for individuals with eating disorders (when recommending diet or physical activity interventions). The focus was on prioritizing patient safety and avoiding potential harm while acknowledging that symptom severity influences patient engagement and motivation regarding physical health interventions. In addition, patients may have limited technology skills or literacy and may need additional support or training on how to use apps [27]. Promisingly, previous research [28,29] has shown that individuals who are less familiar with DLIs can use them after minimal training or with support from peer coaches. Incorporating peer coaches to train and support patients could help lighten the workload burden on MHCPs and contribute to reducing the digital divide [30].

#### Opportunity

Competing interests and limited time, limited patient contact, and inadequate technology infrastructure in the NHS were perceived as barriers to implementing DLIs in MHC settings for MHCPs. This was in line with a recent study that found that MHC settings lacked effective integration of digital technologies [31]. The study also found significant differences among MHCPs regarding whether it falls under their role to implement digital MHC [31]. Therefore, ensuring successful integration of DLIs in MHC requires a thoughtful evaluation of their alignment with existing services, restrictions in inpatient settings, the optimal MHCP role or roles to deliver DLIs, and ways to minimize unnecessary burden on MHCPs.

One solution alongside improvements in technology infrastructure is new MHCP roles dedicated to implementing DLIs and monitoring patient data in MHC settings via the digital navigator pathway [30]. In addition to alleviating burdens, digital navigators could provide feedback to patients on their data. Berry et al [32] discovered that patients expressed a keen interest in engaging with MHCPs to review digitally collected outcome data related to their mental health. This notion was mirrored in this study, with MHCPs emphasizing the need for feedback on health data to gain deeper insights into causal links between behavior and health or to underscore the effectiveness of subtle changes that might take time to be realized more broadly. Although concerns were expressed about collecting and monitoring health data, there are several benefits to using near–real-time data—they can improve the quality of care received, detect health deterioration earlier, and reduce staff demands through more efficient monitoring [33-35].

In contrast with other research exploring MHCPs’ views on digital health for the self-management of severe mental health conditions [32], we found that access to phones was not perceived as a barrier, likely due to the young age of the patient population (18-35 years) and increase in mobile phone ownership in the last decade [36]. However, limited data and app subscriptions were perceived as significant barriers. Staff felt that the NHS should provide phones and wearables and cover the cost of app subscriptions and data allowance. If the NHS is to cover app subscriptions and data allowance, the cost-effectiveness of DLIs needs to be considered to reduce the digital divide [30,37,38].

#### Motivation

In line with previous research, staff were reluctant to promote physical health [10,39,40] because it was perceived as not part of their role. MHCPs also lacked confidence in delivering DLIs and perceived that young people with mental illness have other priorities than their physical health and that risk of harm to patients may outweigh potential benefit. This perceived distinction between physical and mental health needs to be addressed through wider changes in education or service provision to highlight the need to take a holistic approach and treat mental and physical health concurrently [10]. Staff were particularly reluctant to implement DLIs for young people in inpatient settings, where the environment may be more unpredictable and access to technologies may be restricted in some cases. Furthermore, MHCPs felt that young people may be difficult to engage in this environment due to feeling out of control and to restrictions on movement [41]. However, introducing DLIs may provide patients with the autonomy to look after their own physical health when they feel out of control [42] as well as the potential therapeutic effects from physical activity [43]. To address MHCPs’ concerns regarding professional liability and patient safety, clear guidelines and frameworks will need to be in place. MHCPs also stressed the significance of engaging both MHCPs and patients in the development and implementation of DLIs in MHC, believing that this collaborative approach can enhance overall buy-in from MHCPs and patients alike.

### Strengths and Limitations

A strength of this research is the potential real-world impact on patient care by providing a rich understanding of MHCP experiences and clinical recommendations to implement digital health in MHC settings. Our sample comprised primarily White British and female individuals; this means that the findings may not be transferable across cultures, gender, and ethnicities. As with all qualitative research, this study was shaped by the researchers’ personal experiences and views stemming from their own use of digital health and views on DLIs. A reflexive approach among the research team reflecting on the researchers’ personal experiences, assumptions, and perspectives was used throughout the research process.

### Recommendations for Implementation

We make 5 recommendations based on our findings. First, clear guidelines for recommending apps, handling data, and monitoring safety are needed. These guidelines should be developed with stakeholders, patients, and MHCPs to ensure that their concerns and needs are addressed. Second, DLIs should be coproduced, to some extent, with their intended end users to ensure that they are appropriate, engaging, and user-friendly and with those who will be delivering the DLIs to ensure that MHCPs have the skills and these steps may lead to better engagement. While coproduction of DLIs could involve full cocreation of bespoke technologies, this may not always be required, and instead, coproducing the way in which an existing technology is implemented in MHC and provided to patients could be sufficient. Third, to boost MHCPs’ psychological capability and skills in using digital technology, training should be provided. This training could be provided by colleagues who were involved in implementing DLIs to gain clinician buy-in or as part of their professional training, which could lead to increased adoption, better implementation, and improved outcomes for patients. Alternatively, the delivery of this training by patients could help dispel certain preconceived notions, such as the belief that service users may be unable or unwilling to effectively use apps. Fourth, MHCPs need to be made aware of evidence-based digital resources available to them. Finally, a patient-centered approach should be used when implementing DLIs that considers patients’ current symptomatology, particularly regarding paranoia, and clear criteria should be set to reduce any potential risk.

### Future Research

Areas for future consideration toward real-world implementation are (1) the provision of apps, data, or phones to reduce the digital divide; (2) the practical, ethical, and security issues regarding the collection and monitoring of health data; and (3) which MHCP role would be best to implement DLIs or whether a new role is required. First, bridging the digital divide is crucial, and we need to ensure equal opportunities for those with mental health conditions to engage with DLIs. Therefore, future work needs to determine the preventative cost and impact of providing DLIs in MHC settings, focusing on patients’ safety, efficacy, the quality of care received, and the impact on patients’ physical health and their use of other health services. Second, it is important to determine the parameters for data collection. This includes working to determine the optimal type and frequency of health or behavior data and how this can be applicable beyond research purposes and actually become clinically useful for improving patient outcomes. In addition, there is a need to address the ethical considerations surrounding data access in clinical settings, ensuring that patients are able to provide informed consent and outlining what happens when patients lose the capacity to provide informed consent. Finally, further efforts are required to establish whether MHCPs can adequately deliver DLIs in their current roles or whether the development of a new role is necessary to maximize the effectiveness of DLIs in MHC settings. In summary, future research is required to examine the sustainability and cost-effectiveness of the implementation of DLIs, the optimal times and means for introducing DLIs to patients, and how this can be tailored to suit the individual needs of people diagnosed with a mental health condition.

### Conclusions

Implementing lifestyle interventions for individuals with SMI is imperative, and the incorporation of DLIs may overcome some of the barriers faced by in-person interventions. Alongside this, implementation during the early intervention period could present a pivotal opportunity for timely approaches to preventing physical comorbidities from arising. The findings from this study suggest that, while digital health has the potential to enhance MHC and the quality of care that patients receive, there are important concerns that MHCPs hold that need to be addressed when considering implementation. Efforts are required to work with patients, MHCPs, and other stakeholders to identify appropriate content and delivery of DLIs, along with the types and content of training required to facilitate their implementation in routine clinical practice.

## Appendix

Multimedia Appendix 1 COREQ (Consolidated Criteria for Reporting Qualitative Research) checklist.

Multimedia Appendix 2 Interview schedule.

Multimedia Appendix 3 Theme summary table.

## Abbreviations

COM-B: Capability, Opportunity, and Motivation–Behavior
COREQ: Consolidated Criteria for Reporting Qualitative Research
DLI: digital lifestyle intervention
MHC: mental health care
MHCP: mental health care professional
NHS: National Health Service
SMI: severe mental illness

## Data Availability

Data sharing is not applicable to this paper as no data sets were generated or analyzed during this study.
