# Supplementary material for: Digital Lifestyle Interventions for Young People With Mental Illness: A Qualitative Study Among Mental Health Care Professionals
Source: JMIR Hum Factors. 2024 Jun 5;11:e53406. doi: 10.2196/53406 (PMC11187511; doi:10.2196/53406)
Supplement: Multimedia Appendix 1 [file humanfactors_v11i1e53406_app1.docx]

**Supplementary file 1 Consolidated criteria for reporting qualitative studies (COREQ): 32-item checklist**

| **No. Item** | **Guide questions/description** | **Reported on Page #** |
| --- | --- | --- |
| **Domain 1: Research team and reﬂexivity** |  |  |
| *Personal Characteristics* |  |  |
| 1. Inter viewer/facilitator | Which author/s conducted the interview? | Page 5 / paragraph 1  (Line 116) |
| 2. Credentials | What were the researcher’s credentials? E.g. PhD, MD | Page 6 / paragraph 2  (Line 148) not specific on page but both have a PhD. |
| 3. Occupation | What was their occupation at the time of the study? | Page 6 / paragraph 2  (Line148) |
| 4. Gender | Was the researcher male or female? | Page 6 / paragraph 2  (Line 151) |
| 5. Experience and training | What experience or training did the researcher have? | Page 6 / paragraph 2  (Lines 149-150) |
| *Relationship with participants* |  |  |
| 6. Relationship established | Was a relationship established prior to study commencement? | Not reported on page  **No** |
| 7. Participant knowledge of the interviewer | What did the participants know about the researcher? e.g. personal goals, reasons for doing the research | Page 5 / paragraph 1  (Line 119-124)  Participants were briefed on the purpose of the study and written informed consent was obtained |
| 8. Interviewer characteristics | What characteristics were reported about the inter viewer/facilitator? e.g. Bias, assumptions, reasons and interests in the research topic | Page 6 / paragraph 2  (Lines 150-153, 156-156) |

| **Domain 2: study design** |  |  |
| --- | --- | --- |
| *Theoretical framework* |  |  |
| 9. Methodological orientation and Theory | What methodological orientation was stated to underpin the study? e.g. grounded theory, discourse analysis, ethnography, phenomenology, content analysis | Page 4 / paragraph2  (Lines 102-104) |
| *Participant selection* |  |  |
| 10. Sampling | How were participants selected? e.g. purposive, convenience, consecutive, snowball | Page 5 / paragraph 2  (Lines 120-123) |
| 11. Method of approach | How were participants approached? e.g. face-to-face, telephone, mail, email | Page 5 / paragraph 2  (Line 121) |
| 12. Sample size | How many participants were in the study? | Page 7 / paragraph 2  (Line 178) |
| 13. Non-participation | How many people refused to participate or dropped out? Reasons? | **Not reported in paper - None** |
| *Setting* |  |  |
| 14. Setting of data collection | Where was the data collected? e.g. home, clinic, workplace | Page 5 / paragraph 3 (Line 127) & Page 29 / paragraph 5  (Lines 229-233)  . |
| 15. Presence of non-participants | Was anyone else present besides the participants and researchers? | **No** |
| 16. Description of sample | What are the important characteristics of the sample? e.g. demographic data, date | Page 7 / paragraph 2  (Lines 178-182) |
| *Data collection* |  |  |
| 17. Interview guide | Were questions, prompts, guides provided by the authors? Was it pilot tested? | Supplementary file 2; page 5 / paragraph 3 (Lines 128-129 & 100) |
| 18. Repeat interviews | Were repeat inter views carried out? If yes, how many? | **No** |
| 19. Audio/visual recording | Did the research use audio or visual recording to collect the data? | Page 5 / paragraph 3 (Line 127) |
| 20. Field notes | Were ﬁeld notes made during and/or after the inter view or focus group? | No |
| 21. Duration | What was the duration of the inter views or focus group? | Page 5 / paragraph 3 (Line 128) |
| 22. Data saturation | Was data saturation discussed? | No- but we stopped the interviews when no new ideas emerged from the last 3 interviews. |
| 23. Transcripts returned | Were transcripts returned to participants for comment and/or correction? | **No** |
| **Domain 3: analysis and ﬁndings** |  |  |
| *Data analysis* |  |  |
| 24. Number of data coders | How many data coders coded the data? | **One (C.S)** |
| 25. Description of the coding tree | Did authors provide a description of the coding tree? | No |
| 26. Derivation of themes | Were themes identiﬁed in advance or derived from the data? | Page 6 / paragraph 2 (Lines 153-155)  A primarily inductive approach was adopted with the interviews but a deductive approach was taken when looking at the barriers and facilitators informed by the COM-B model (based on previous research). |
| 27. Software | What software, if applicable, was used to manage the data? | Page 6 / paragraph 2 (Line 157) |
| 28. Participant checking | Did participants provide feedback on the ﬁndings? | **No** |
| *Reporting* |  |  |
| 29. Quotations presented | Were participant quotations presented to illustrate the themes/ﬁndings? Was each quotation identiﬁed? e.g. participant number | Yes Page 8-10 Table 1 |
| 30. Data and ﬁndings consistent | Was there consistency between the data presented and the ﬁndings? | **Yes** |
| 31. Clarity of major themes | Were major themes clearly presented in the ﬁndings? | Page 7 / paragraph 3 (Lines 185-186) ~ Page 18 / paragraph 1 (Line 214)  **Yes** |
| 32. Clarity of minor themes | Is there a description of diverse cases or discussion of minor themes? | **Yes** Page 8-10 Table 1 |
