# Supplementary material for: Digital Lifestyle Interventions for Young People With Mental Illness: A Qualitative Study Among Mental Health Care Professionals
Source: JMIR Hum Factors. 2024 Jun 5;11:e53406. doi: 10.2196/53406 (PMC11187511; doi:10.2196/53406)
Supplement: Multimedia Appendix 2 [file humanfactors_v11i1e53406_app2.docx]

Supplementary material 2. Semi-Structured Interview Schedule

**Semi-Structured Interview Schedule**
+ Optional discussion points

***Part 1 – Background/Overview***

*[Begin with Introductions, overview of the project (including asking if they participated in Survey), and outlining what these qualitative interviews will be about]*

1. **Can you tell me about your current role?**

+ With particular reference to working with Young Adults (i.e. Aged 16 – 30) with Mental Illness

**2. Do you have any professional experience of using mHealth or digital health in your role as a healthcare professional?**
+ e.g.s include health apps, recording or using digital data, even Zoom/video conferencing with patients?
+ If ‘yes’, ask for their thoughts on these

**3. Do you have any personal experience of using mHealth or apps for your own health/fitness?**
+ e.g.s include Apps for meditation, exercise, smoking, diet? Fitbit or wearable devices?
+ If ‘yes’, ask for their thoughts on these

***Part 2: Patient-related Factors***

1. **In what ways could apps or mhealth be used for promoting physical health in young people with mental illness?**

+ Monitoring Physical Health and Weight gain?

+ Promoting Exercise / Fitness?

+ Quitting Smoking?

+ Tracking Sleep?

+ Helping with Diet?

+ Any other ideas?

1. **What barriers might prevent the use of apps/mhealth in this group?**

+ How to overcome these

+ What types of patients might benefit/be left out?

1. **How could we promote engagement with apps among young adults with mental illness?**

+ Could apps engage patients?

+ What would motivate people? Payments? Tracking/Sharing Data? Clinician feedback? Fun/gameified apps?

***Part 3: Professional-related Factors***

**1. Which kind of Healthcare Professional (HCP) would be best placed to recommend/’prescribe’/provide patients with apps for promoting physical health?**
+ What should their role be? Just recommending? Showing them around the apps? Installing? Linking? Etc…

**2. What are the barriers for HCPs towards using health apps in the care of young adults with mental illness?**
+ E.g. time/capacity, knowledge, motivation, capability
+ Any concerns? E.g. doing more harm, not being secure, invading patients privacy etc.

**3. What tools or training would be useful for HCPs to enable them to provide app-based physical health interventions for young adults with mental illness?**
+ How can we make mental health care workers feel “comfortable”/ increase confidence?
+ What would they want from training (duration, frequency, content [do they want a demonstration or to know evidence behind effectiveness], mode of delivery [remote/in person])

***Part 4: Tech-related Factors***

**From a technological perspective, what do you see as the main opportunities or issues for integrating physical health apps into mental health care?**E.g.s:

+ Using technologies for supporting digital data collection / clinical health monitoring? (along with who should/could access data etc?)

+ Integration new tech/apps with current physical health initiatives in healthcare? Would adding wearables be feasible/useful?

+ Would using digital tech interfere with face-to-face care, or enhance it?

**Final**

Is there anything else you would like to add that you feel we have not already covered?
