# Supplementary material for: Digital Lifestyle Interventions for Young People With Mental Illness: A Qualitative Study Among Mental Health Care Professionals
Source: JMIR Hum Factors. 2024 Jun 5;11:e53406. doi: 10.2196/53406 (PMC11187511; doi:10.2196/53406)
Supplement: Multimedia Appendix 3 [file humanfactors_v11i1e53406_app3.docx]

**Table 1.** Theme summary.

| Theme and subtheme | | Description | Example quote(s)^a^ | Link to COM-B^b^ model |
| --- | --- | --- | --- | --- |
| **Motivation will affect implementation** | | | | |
|  | 1. Individuals are motivated, but others are resistant | Interviewees discussed MHCPs’^c^ resistance to change despite their own personal positive attitude toward digital health. | - “...you do get a lot of staff who will say, well we’ve always done it like this, yeah, we know you’ve always done it like this.” [INT5] | - Opportunity—social: beliefs about other motivations |
|  | 2. Patients have other priorities | Interviewees perceived that patients prioritize activities to promote positive mental health rather than their physical health. | - [physical health] “It it’s not the patient’s priority. So it’s, it’s not, it’s not a concern for them.” [INT4] - “...if people have, if people’s lives are sort of a little bit chaotic or, it’s you know, sort of unpredictable...it just wouldn’t be on your mind, other priorities would take over.” [INT9] | - Motivation—reflective: service users have low motivation |
|  | 3. DLIs^d^ need to be intuitive and engaging | Interviewees talked about how digital platforms should be easy to use/implement and engaging to boost motivation to use DLIs for patients and MHCPs. | - “I’m not particularly interested in gamified devices, I just want (laughs) I just want cold hard data on what I’ve done and, erm, but I, you know, I could see my son, who’s a teenager, that’d be much more appealing for him to have something that was a bit more fun to use and, erm, you know might, that might be a bit more engaging.” [INT1] - “You know, more colourful, more upbeat, more interactive, erm, different things going on, erm, that people can access.” [INT10] - “...the important thing would be that if you were gonna design an app to, to genuinely have young people with a variety of mental health conditions, neurodevelopmental disorders, etc, all having input on what it looks like, how it works and how you engage with it.” [INT13] | - Motivation—automatic: if it is engaging and easy to use, it will encourage engagement with DLIs |
| **Patients’ readiness and capability** | | | | |
|  | 1. Patients’ safety | Patient safety was frequently mentioned. It included risk if MHCPs missed important data and making sure apps were safe to use and did not cause harm to patients through exacerbating symptoms of paranoia, following exercise videos, by providing misinformation, or as a vulnerable group being taken advantage of. | - “...young people in inpatient settings people become very, perhaps over focused on tracking health outcomes, tracking weight, tracking calories, erm, tracking how many calories you’re burning off, and you know, particularly if people might have eating disorders or difficulties like that, you might actually compound their difficulties rather than help their difficulties.” [INT9] | - Motivation—reflective: concerns about patient safety |
|  | 1. Patients’ capability and opportunity to use digital technology | Patients’ technology skills, psychological capability diminished by poorer mental health, and literacy were mentioned as perceived barriers to engaging with DLIs. Patients’ access to smartphones, digital poverty, and affordability and availability of data were perceived barriers to engaging with DLIs. | - “...in our kind of population, we have some patients that English isn’t their first language, and the processing skills to navigate the app, that would be a bit of a challenge I think for a few.” [INT8] - [potential barrier] “just being able to, navigate it, erm, but if it was set up I suppose, that’d be easier. I do get a lot of service users in my previous role coming in asking me to set things up for them on the phone, whether that be banking apps or something like that, so maybe just the ability to do that as well.” [INT12] - “The vast majority of young people have a phone, and have a phone that they can download apps on.” [INT13] - “...a lot of people do have phones, but sometimes they don't. So we do our patients who don't have a phone at all we have patients, especially when they first come in a very chaotic. They've lost their phone...within the hospital they get access to the free Wi-Fi, which is great. And so it wouldn't be an issue there, but they may not have Wi-Fi at home.” [INT2] | - Capability—psychological: not having the skills to use DLIs - Opportunity—physical: not having access to data or phones |
| **Reallocation of staff roles and responsibilities** | | | | |
|  | 1. Technology changes our roles and responsibilities | Interviewees perceived that implementing DLIs would change their roles, workloads, and responsibilities. | - “...the whole digital health area feel like it’s moving away from what, what people see as the sort of the core function of mental health clinicians and, and professionals which is around sort of forming meaningful relationships with people.” [INT9] - “...if they were recording their weight in stuff like that, that would be amazing for us, because, I think like NICE guidance, especially when somebody comes into, erm, like a psychosis service, they’re supposed to be actually weighed weekly for like the first six weeks, but, I don’t know anywhere that does that, erm, so, I think things like that would be amazing...anything that they can input for us, even if they stop smoking, or start smoking, you know that would be amazing for us to know, ’cos we need to know that every year.” [INT10] - “I don’t like the idea about it feeding in, I prefer the idea of, I think it’s a fantastic idea for them to go away and use the, erm, use the systems in real time, so when they come back and you say, well go on, your weight, you’ve been putting weight on, you say you’ve not, let’s have a look on your app what you actually have been eating, so you can have those sort of discussions with them, erm, no I don’t like the idea of the feeding into digital systems and getting no, no human feedback.” [INT6] - “I think it’d be great to just say, you know, once you’re in our service we’d offer this app and you know it can help you kind of live a healthier lifestyle and it has, you know, lots of different information on it, lots of different support information, you know, we will, it will kind of send important, certain data to us about you know, your, kind of height and weight which is really important to us, ‘cos we’re monitoring your medication, so it saves you having to keep coming in.” [INT10] | - Opportunity—physical: time constraints and integrating DLIs into their roles |
|  | 2. MHCPs will need to acquire additional skills | MHCPs lack knowledge about and skills to introduce and deliver DLIs and, therefore, need training in how to implement DLIs in the mental health care setting. | - “I think if you wanted all the staff to support and facilitate using an app to monitor physical health, I think in mental health to be honest, for some people you’d probably need some basic education on why it’s important...nd then also some training on just how to use the app, ’cos I think for a lot of people who work in mental health they aren’t always confident using technology.” [INT11] - “I think it would be really helpful to have training on how it works, how people can access it, like how we, how we should be explaining it to people, and like ways that we can use it to enhance our working with them.” [INT13] - “It would depend how complex the app is, wouldn't it? I suppose because sometimes with apps like if you could just do a little YouTube video of this is how use this, but this is how you use this bit. But if it's going to be more complicated and in putting things in, you might need some sort of face to face training.” [INT2] | - Capability—psychological: MHCPs’ skills to use the digital systems |
|  | 3. Who is responsible for managing the risk? | Due to perceived risk to patients, liability and risk of harm were huge concerns, which included discussions about monitoring of data, what data should be monitored and by whom, and the risks if data are not monitored. | - “...you’ve got a patient with mental health difficulties whose got motivation issues, you know, difficult to engage...they get so confused over the smallest thing...no I don’t like the idea about it” [data] “feeding in, I prefer the idea of, I think it’s a fantastic idea for them to go away and use the, erm, use the systems in real time, so when they come back and you say, well go on, your weight, you’ve been putting weight on, you say you’ve not, let’s have a look on your app what you actually have been eating.” [INT6] - “...we’ve got to think about the governance, so if somebody’s got, erm, weight that’s increased over a certain space of time, who’s role and responsibility is it gonna be to pick that up and do something with it.” [INT5] | - Opportunity—physical - Motivation—reflective: beliefs about data security and concerns about patient safety |

^a^Some quotes are also presented in the main text.

^b^COM-B: Capability, Opportunity, and Motivation–Behavior.

^c^MHCP: mental health care professional.

^d^DLI: digital lifestyle intervention.
